# MyD88 and beyond: a perspective on MyD88-targeted therapeutic approach for modulation of host immunity

**DOI:** 10.1007/s12026-021-09188-2

**Published:** 2021-04-08

**Authors:** Kamal U. Saikh

**Affiliations:** Department of Bacterial Immunology, Bacteriology Division, United States Army Medical Research Institute of Infectious Diseases, 1425 Porter Street, Frederick, MD 21702 USA

**Keywords:** MyD88, TLRs, SEB, Sepsis, LPS, Poly I: C, IFN-β

## Abstract

**Abstract:**

The continuous emergence of infectious pathogens along with antimicrobial resistance creates a need for an alternative approach to treat infectious diseases. Targeting host factor(s) which are critically involved in immune signaling pathways for modulation of host immunity offers to treat a broad range of infectious diseases. Upon pathogen-associated ligands binding to the Toll-like/ IL-1R family, and other cellular receptors, followed by recruitment of intracellular signaling adaptor proteins, primarily MyD88, trigger the innate immune responses. But activation of host innate immunity strongly depends on the correct function of MyD88 which is tightly regulated. Dysregulation of MyD88 may cause an imbalance that culminates to a wide range of inflammation-associated syndromes and diseases. Furthermore, recent reports also describe that MyD88 upregulation with many viral infections is linked to decreased antiviral type I IFN response, and MyD88-deficient mice showed an increase in survivability. These reports suggest that MyD88 is also negatively involved via MyD88-independent pathways of immune signaling for antiviral type I IFN response. Because of its expanding role in controlling host immune signaling pathways, MyD88 has been recognized as a potential drug target in a broader drug discovery paradigm. Targeting BB-loop of MyD88, small molecule inhibitors were designed by structure-based approach which by blocking TIR–TIR domain homo-dimerization have shown promising therapeutic efficacy in attenuating MyD88-mediated inflammatory impact, and increased antiviral type I IFN response in experimental mouse model of diseases. In this review, we highlight the reports on MyD88-linked immune response and MyD88-targeted therapeutic approach with underlying mechanisms for controlling inflammation and antiviral type I IFN response.

**Highlights:**

• Host innate immunity is activated upon PAMPs binding to PRRs followed by immune signaling through TIR domain–containing adaptor proteins mainly MyD88.

• Structure-based approach led to develop small-molecule inhibitors which block TIR domain homodimerization of MyD88 and showed therapeutic efficacy in limiting severe inflammation-associated impact in mice.

• Therapeutic intervention of MyD88 also showed an increase in antiviral effect with strong type I IFN signaling linked to increased phosphorylation of IRFs via MyD88–independent pathway.

• MyD88 inhibitors might be potentially useful as a small-molecule therapeutics for modulation of host immunity against inflammatory diseases and antiviral therapy.

• However, prior clinical use of more in-depth efforts should be focused for suitability of the approach in deploying to complex diseases including COPD and COVID-19 in limiting inflammation-associated syndrome to infection.

## Introduction

**Fig. 1:**
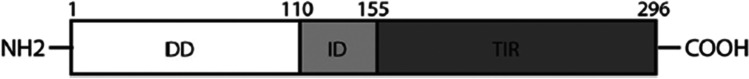
MyD88 has a modular structure composed of three main domains: an N-terminal death domain (DD) (54 to 109), intermediate domain (INT) (110 to 155), and Toll-interleukin-1 receptor domain (TIR) (155 to 296)

Host sense variety of danger signals including exposure to microbial pathogens or pathogen-associated molecular patterns (PAMPs) using a family of innate immune receptors, known as pathogen recognition receptors (PRRs) such as Toll-like receptors (TLRs), the retinoic acid-inducible (RIG-I)–like receptors (RLRs)/melanoma differentiation–associated gene-5 (MDA-5), and C-type lectin receptors [1–5]. Most of these pathogens are recognized by ligand (PAMPs) binding to more than one class of these innate immune receptors [1–5] and trigger diverse innate signaling pathways which initiate a range of host defense mechanisms. In mammals, there are more than 10 members of the TLRs that recognize conserved components of microorganisms or PAMPs. TLR(s) initiated activation of innate immune signaling cascades lead to induction of inflammatory responses with subsequent development of antigen-specific adaptive immunity. Most of the TLR-initiated inflammatory responses except TLR3 are dependent on a common signaling pathway that is mediated by the recruitment of intracellular signaling adaptor proteins including myeloid differentiation primary response protein (MyD88), which associates with the MyD88 adaptor like (Mal, also known as Toll-interleukin receptor domain (TIR) containing adaptor protein or TIRAP) and activates pro-inflammatory cascades [6]. MyD88 was first identified in 1990 as a gene activated in M1D + myeloid precursors, following induction of terminal differentiation and growth arrest by IL-6. The full-length cDNA sequence and the amino-acid sequence of MyD88 were deduced [7]. MyD88 RNAs were also detected in myeloid precursor enriched murine bone-marrow, but not in non-myeloid murine tissues. Later, Muzio et al. and Wesche et al. discovered that MyD88 is a proximal signaling adaptor of the IL-1R signaling pathway and demonstrated that MyD88 transduces IL-1R1 initiated signaling to the transcription factors NF-kB [8, 9]. In co-immunoprecipitation experiments, it was shown that the TIR domain of MyD88 interacts with the TIR domains of IL-1R1 and IL-1RAP. Besides MyD88, another four TIR-containing adaptor molecules involved in TLR-IL-R signaling networks have been identified. These include MyD88-adaptor–like (Mal, also known as TIR domain–containing adaptor protein or TIRAP), TIR domain–containing adaptor protein inducing interferon-β (TRIF), TRIF-related adaptor molecule (TRAM), and sterile-a-and-armadillo motif-containing protein (SARM) [5–17]. Recruitment of these TIR domain–containing adaptors to the TIR domains of TLRs is a critical framework to transmit a signal to the nucleus and also requires different adaptor proteins for transduction of signals by TLR/IL-IRs [18, 19]. These adaptor proteins engage the signaling cascade of protein kinases that trigger activation of transcription factors and expression of genes involved in immune response, such as IL-12, IL-6, IL-8, IL-1β, and IFNs [20]. Among the various TLR-IL-1R adaptor proteins, MyD88 functions as the conventional partner. MyD88 is required for the activation of MyD88-dependent signaling pathways specific to all TLRs except TLR3 as well as for stimulation of cells by IL-1, IL-18, or IL-33 [21]. MyD88 has a modular structure composed of three main domains: a death domain (DD) and a TIR domain that are separated by an intermediate segment or intermediary domain (INT) [22]: an N-terminal death domain (DD) comprises (54 to 109), intermediate domain (INT) (110 to 155), and Toll-interleukin-1 receptor domain (TIR) (155 to 296) (Fig. 1). The TIR domain of MyD88 located at its C terminus is responsible for binding to the receptor TIR domain [23], whereas the N-terminal DD is responsible for binding to the IL-1R–associated kinase (IRAK) 4 and for further propagation of the signal in the signaling pathway [24]. The absence of INT has been associated with the inability of MyD88 to support signaling [25]. Based on the unique structure, MyD88 serves as the central link that connects TLR-IL-1R family members to IL-R-associated kinases (IRAKs). MyD88 binds the serine–threonine kinases IRAK1 and IRAK2, mammalian homologs of Drosophila Pelle in the Toll pathway, via a heterotypic death domain–mediated interaction and thus acts as a pure adaptor linking the IL-1R1 to downstream IRAK kinases. Thus, central to MyD88 function is the ability of its TIR domain to heterodimerize with the TIR domain of the receptor and homodimerize with another MyD88 molecule to recruit downstream signaling molecules. So far, it has been established that the MyD88 TIR domain and DD can on their own inhibit MyD88-dependent pathways [26], whereas expression of DD linked with INT leads to the constitutive activation [26, 27]. Recently, the crystal structure of the complex of DDs of MyD88–IRAK4–IRAK2 (segment of Myddosome) [28] was resolved, demonstrating the formation of a large complex with stoichiometry 6:4:4. A conserved sequence, (F/Y)-(V/L/I)-(P/G), called the BB-loop appears in the TIR domain of most members of the TLR/IL-1R family. The BB-loop is reported to be involved in TIR-TIR interaction and is critical in MyD88-mediated inflammatory signaling [29].

### Beside TLR/ IL-1Rs, involvement of MyD88 in other intracellular inflammatory signaling

MyD88 was originally known to be involved in signal transduction from TLR and IL-1R family members with exception of TLR3 [6, 17, 20, 29, 30]. In 2006, Sun and Ding reported that MyD88 is also involved in transmitting signals induced by interferon-γ (IFN-γ) in macrophages and demonstrated that MyD88 deficiency results in diminished production of TNF-α- and IFN-γ-inducible protein 10 (IP-10) treated with IFN-γ [31]. These results demonstrated that the involvement of MyD88 goes beyond the signal transduction process other than TLR-IL-1R family members. Subsequently, Liu et al. reported that the deficiency in MHC class II resulted in impaired TLR triggered production of pro-inflammatory cytokines and protected mice from an otherwise lethal challenge with TLR ligands and live Gram-negative bacteria [32]. This study also concluded that both the TLR- and MHC-mediated responses engage MyD88 [33]. MHC class II molecules are known to activate various cellular functions in immune and non-immune cells when cross-linked by antibody or superantigens [34–36]. Staphylococcal enterotoxins (SEs) including SEB also known as superantigens, upon exposure through non-enteric route such as inhalation, cause a life-threatening toxic shock syndrome (TSS). The profound clinical consequences of SEB-induced TSS often leads to organ failure and death which are due to an excessive pro-inflammatory cytokine response such as tumor necrosis factor-alpha (TNF-α), interferon-gamma (IFN-γ), interleukin-6 (IL-6), and interleukin-1beta (IL-1β) [37, 38]. SEB binding to MHC class II molecules on antigen-presenting cells and cross-linking to T cell receptors triggered the event. The results from our laboratory demonstrated that MyD88 gene–knockout mice (MyD88^−/−^) were resistant to staphylococcal enterotoxin A (SEA) [39] or SEB-induced toxic shock [40] and showed a reduced level of pro-inflammatory cytokines in serum. In contrast, the potent cytokine response of wild-type mice was significant and lethal. It has also been demonstrated that SEA or SEB exposure activated MHC class II-linked MyD88-mediated pro-inflammatory cytokine signaling in human monocytes [41]. In human primary cells, MyD88 gene silencing using siRNA followed by SEB or SEB plus LPS stimulation results in decreased transcriptional activation of MyD88 and lower expression of IL-1β [42]. These data validate that in addition to TLR-IL-1R family of receptors, MyD88 is also an essential signaling component engaged in SEB-induced pro-inflammatory cytokine responses.

### MyD88 as a therapeutic target for intervention of severe inflammatory response

**Fig. 2:**
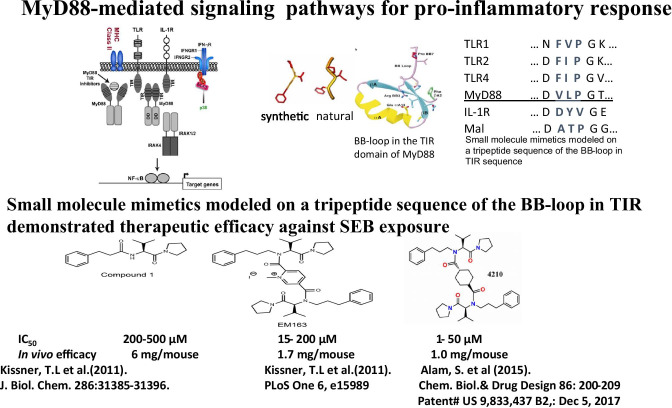
Schematic representation of MyD88-mediated pro-inflammatory signaling, design of small molecules based on BB-loop structures in TIR domain of MyD88 that showed therapeutic efficacy in mice against SEB intoxication

While recruitment of MyD88 is a prerequisite for inflammatory signaling and convergence point of multiple pro-inflammatory cytokines, dysregulation or overstimulation leads to inflammation-associated syndromes with severe pathological consequences to host; thus, MyD88 appears to be a unique target for therapeutic intervention of severe pro-inflammatory cytokine signaling. It has been shown that the BB-loop region acts as the mediator of the homo (adaptor-adaptor)- and hetero (receptor-adaptor)dimerization that is necessary for the function of TIR domains to induce MyD88-mediated signaling [6, 17, 43]. Bartfai et al. showed that a synthetic molecule, hydrocinnamoyl-L-valyl-pyrrolidine, (AS1), mimicking the BB-loop of the TIR domain, representing consensus the amino acid sequence of RDVLPGT (aa196-202) disrupted TLR/IL-1R signaling as shown in Fig. 2 [44]. The mimetic blocked IL-1 signaling by disrupting MyD88 and IL-1R association and reduced fever associated with inflammation in mice. By application of structure-based approach a small molecule compound1 mimicking the BB-loop of TIR domain of MyD88 was initially designed which did interfere MyD88-mediated signaling (Fig. 2) [42]. Later, a modified dimeric compound EM-163 synthesized from compound1 joined by an aromatic benzene ring and found to be more effective than compound I which attenuated TNF-α, IFN-γ, IL-6, IL-2, and IL-1β in human primary cells with exposure to SEB. Pretreatment of EM-163 completely abrogated TNF-α, IFN-γ, IL-6, IL-12p70, and IL-1β and protected BALB/c mice from toxic shock–induced deaths from lethal SEB challenge and remained healthy [45]. EM-163 treatment also protected mice from post-exposure to SEB. Also, pretreatment of EM-163 to C57BL/6 mice also completely abrogated TNF-α, IFN-γ, IL-6, IL-12p70, and IL-1β responses and protected from SEA lethal challenge. Furthermore, modification of EM-163 was made utilizing compound **1** [42, 46] by covalent linkage using nonpolar cyclohexane [47, 48]. Dimeric compound 4210 was synthesized in which two modules of compound **1** were covalently linked by a non-polar cyclohexane ring so that a six-member heterocycle ring will increase flexibility for binding to the exposed BB-loop of the TIR domain to interact with the target domain of MyD88. Compound 4210 strongly inhibited the production of pro-inflammatory cytokines in human primary cells to SEB or LPS extracted from *Francisella tularensis*, *Escherichia coli*, or *Burkholderia* *mallei*. Consistent with cytokine inhibition, in a ligand-induced cell-based reporter assay, compound 4210 inhibited *B.* *mallei* or LPS-induced MyD88-mediated NF-kB-dependent expression of reporter activity. Furthermore, results from a newly expressed MyD88 revealed that dimeric compound 4210 inhibits MyD88 dimer formation which is critical for pro-inflammatory signaling and a single administration of compound 4210 in mice showed complete protection from lethal toxin challenge. BB-loop mimetic inhibited homodimerization of MyD88 through TIR domain interaction and blocked MyD88-mediated signaling [47]. In addition to compound 4210, tri-peptide derivative compound 7, synthesized to mimic a key BB‐loop region involved in (TIR) domain interactions, was a potent, stable, and drug‐like small molecule and was shown to attenuate pro-inflammatory cytokines in human peripheral blood mononuclear cells and bronchial epithelial cells challenged with a live vaccine strain of *F.* *tularensis* [49]. It appears that small molecules which target TIR domain interactions in MyD88‐dependent TLR signaling represent a promising strategy toward host‐directed therapeutics against infection‐induced inflammation-associated sepsis.

Besides, BB-loop mimetic using an additional alternative approach by building a protein–protein dimeric docking model of the TIR-domain of MyD88, Olson et al. identified a binding site for docking small molecules. Computational screening of 5 million drug-like compounds led to identifying a molecule T5910047 that inhibits the TIR-TIR domain interaction and attenuates pro-inflammatory cytokine production in human primary cell cultures [50]. Subsequently, based on the structure similarity, compound T6167923 was identified from the PubChem database which was found to be more potent with improved drug-like properties and capable of inhibiting MyD88 homodimer formation critical for the MyD88-mediated pro-inflammatory signaling and completely protected mice from toxic shock–induced death [50, 51].

Mario M et al. reported a synthetic peptido-mimetic compound (ST2825) modeled after the structure of a hepta-peptide in the BB-loop of the MyD88-TIR domain, which inhibited IL-1R/TLR signaling by interfering with MyD88 homodimerization of the TIR domains and did not affect homodimerization of the death domains. Oral administration of ST2825 dose-dependently inhibited IL-1beta-induced production of IL-6 in treated mice [52]. ST2825 also suppressed B cell proliferation and differentiation into plasma cells in response to CpG-induced activation of TLR9, a receptor that requires MyD88 for intracellular signaling, thus, suggesting that it may have therapeutic potential in the treatment of chronic inflammatory diseases [53, 54]. Van Tassell et al. reported that pharmacologic inhibition of MyD88 in vivo attenuates pathologic left ventricular (LV) dilation and hypertrophy in a mouse model of non-reperfused acute myocardial infarction (AMI) independent of infarction in mouse. Data suggest that pharmacologic MyD88 inhibition protects against pathologic LV remodeling without altering infarct scar formation. The report suggests that MyD88 may be a viable target for pharmacologic inhibition in AMI [54].

A spontaneous and sustained activation of MyD88-mediated via NF-κB signaling is associated with inflammation-induced cancer. Xie et al. reported that TLR/MyD88 signaling may be a therapeutic target for colitis-associated cancer (CAC) intervention and designed a MyD88 inhibitor TJ-M2010-5, which was shown to bind to the TIR domain of MyD88 to interfere with its homo-dimerization, and the TLR/MyD88 signal pathway. In a mouse model of azoxymethane/dextran sodium sulfate (AOM/DSS)—induced (CAC) in combination with TJ-M2010-5 administration, the anti-inflammation-related cancer effect of MyD88 inhibitor was investigated in vivo, and MyD88 inhibitors may be a promising therapeutic modality for treating patients with CAC [55].

A mutation associated with nearly 1/3 of human diffuse large B cell lymphomas (DLBCLs) has been identified within MyD88 DLBCLs bearing MyD88L265P. This mutation correlates with tumor cell proliferation and survival involving spontaneous and sustained activation of NF-κB signaling. Protein–protein docking model and in silico identified MyD88 inhibitor derived small molecule compound T6167923 showed inhibition MyD88 dimer formation and were able to inhibit cell proliferation of DLBCLs bearing the MyD88L265P as measured by iso-thermal shift assay. Future studies defining the molecular mechanism of this mutation with additional human patient tumor isolates will inform and propel development of novel therapeutics to counteract both inflammation as well as tumor formation [56].

Chronic obstructive pulmonary disease (COPD) is characterized by emphysema, small airway remodeling, pulmonary hypertension, and chronic bronchitis. Treatment with corticoids has no effects on COPD. Acute exacerbations triggered by infections play an important pathogenic role in COPD. Recent pandemic with COVID-19 disease associated with SARS-CoV-2 infections causes respiratory failure with severe lung inflammation. Thus, there is a clear unmet medical need that requires effective and safe anti-inflammatory or disease-modifying therapies for these critical conditions which are lacking. The critical role of MyD88 in pro-inflammatory signaling associated with severe inflammation especially in chronic lung diseases such as chronic obstructive pulmonary disease (COPD) is summarized in a recent review [51]. The discovery and development of pharmacological inhibitors of MyD88 signaling suggest MyD88 as a drug target to treat respiratory diseases, which may represent a significant therapeutic progress [51]. The proof of concept that therapeutic targeting of MyD88 may be feasible and first preclinical data are highly promising and open a great opportunity to treat exacerbations of COPD and other chronic respiratory diseases. However, extensive preclinical investigations and risk analyses are required with careful evaluation of reduced host resistance and opportunistic infections. In regard to the COVID-19 disease, the role of MyD88 has yet to be undetermined, although it is known that crushing pro-inflammatory cytokine storm is the main reason for lung pathology. Thus, it has been demonstrated that inhibition of MyD88 led to limit pro-inflammatory cytokines like TNF-α and IL-1β [47]. Since MyD88 is a critical signaling adaptor protein, at the convergence of multiple pro-inflammatory pathways involve in inducing host innate immunity, though speculative, pharmacologic inhibition of MyD88 in TIR blockage may likely provide limiting pro-inflammatory cytokines and possibly alleviate lung-associated damage.

## Role of MyD88 in the regulation of antiviral immunity

Viruses are highly infectious pathogens and the physiological response to virus infection is generally initiated at the cellular level following replication [57]. After virus entry, the infected cell detects the presence of virus replication through any one of a number of pattern recognition receptors (PRRs). These PRRs serve as sentinels for a variety of microbes inside and outside of the cell by physically engaging distinct structures that are shared among different pathogens. PRRs are like TLRs/IL-R via MyD88-TRIF, (RIG-I)/ (MDA-5)–mitochondrial antiviral–signaling proteins (MAVS) axis as well as double-stranded RNA-dependent protein kinase (PKR), the DNA receptor, DAI, and cyclic GMP-AMP synthase (cGAS)-stimulator of interferon genes (STING) axis for cytosolic RNA and DNA, respectively [58, 59]. Further cellular detection of viral infections is also mediated by intracellular PRRs that sense aberrant structures that are often formed during virus replication, such as double-stranded RNA, an intermediate byproduct of viral replication [58]. Engagement of the virus-specific RNA structures culminates in oligomerization of these receptors and mount a concerted cellular innate immune response which is the first and most rapid line of host defense against invading pathogens through activation of networks of innate immune signaling pathways. Activation of this innate immune response responses primarily relies on the synthesis of antiviral innate cytokines such as type I interferon (IFN) and type III IFNs (IFN-λ) and secreted by various cell types [60–62], via activation of downstream transcription factors, most remarkably interferon regulatory factors (IRFs) and nuclear factor kB (NF-kB) [63]. Transcriptional activation of IRFs and NF-kB results in the launch of two general antiviral programs, and subsequent upregulation of IFN-stimulated genes (ISGs). Induction IFNs lead to several antiviral proteins such as dsRNA-activated protein kinase R, 2–5 oligo-adenylate -synthase, and Mx1 protein which ultimately mediate the antiviral actions of IFNs. Due to these strong host-directed innate immune responses, in many instances, viral infections are uneventful, and patients recover and either eliminates the virus or incorporate it into a latent or persistent form without further problems. In order to establish a productive infection, viruses need to evade and overcome this initial anti-viral type I (IFN α/β) also type III IFN (IFN-λ, 1-IV) responses [64]. Type I IFNs in addition to immediate response also promote activation of other cells of the immune system such as dendritic cells, T cells, B cells, and NK cells that enhance adaptive immune response and prevent dissemination of the pathogen and disease progression [65]. Because of its significant role in antiviral defense, type I IFN has been approved for clinical use, when effective antivirals or vaccination strategy are not available. It has also been recommended for the recent COVID-19 pandemic [66].

Although transcriptional factors such as IRFs are the crucial transcription factors that control expression of IFN, with a viral infection, and NF-kB regulates the pro-inflammatory cytokines and type 1 IFNs during microbial infection, other cellular factors such as adaptors (MAVS, TRIF, TRAF3/TRAF6, MyD88) and downstream kinases such as IKK-e/TBK or IKB upstream of IRFs or NF-kB are also important host factors enable transcriptional activation of IRFs and NF-kB. Viral products, such as double-stranded RNA (dsRNA), an important byproduct of replication of many viruses, are recognized by more than one PRRs such TLRs and (RLRs)/ (MDA-5), wherein activation of IRF-3 and IRF-7 is critically involved in the regulation of IFN-α/β gene induction [67–70]. At the early stage of viral infection, type 1 IFN production subsequently triggers antiviral responses by binding to a common factor, the interferon receptor (IFNR). IFN α/β binding to the IFNR stimulates the JAK1-STAT pathway leading to the assembly of the IFN-stimulated gene factor 3 (). ISGF3 complex which is composed of STAT1-STAT2 dimers and IRF9 binds to IFN-stimulated response elements (ISRE) in the promoters of IFN-stimulated genes to regulate their expression and subsequently leads to robust second wave of various ISG expression. Thus, full-fledge activation of host innate immunity through autocrine signaling in the infected cells and paracrine signaling in surrounding uninfected cells creates an antiviral state in surrounding cells. This is possible only with the availability of the IRF-3/IRF-7 without any constraint, and appropriate IRF(s) phosphorylation which is crucial for the induction of first wave of strong IFN-I response [67, 71–74]. However, sequestration of IRFs would limit IRF phosphorylation resulting in weak IFN-I signaling and insufficient IFN-I output needed for strong antiviral immunity in the host [73, 74].

Host adaptor proteins play a critical role with TLR pathway of immune signaling in inducing type 1 IFN response, and particularly MyD88 is used by all TLRs, except TLR3, and is shared by IL-1 and IL-18 receptors, while TIR domain–containing adaptor-inducing IFN-β (TRIF) is solely engaged by TLR3 and TLR4. Furthermore, while recruitment and activation of the adaptor proteins MyD88 or TRIF are critical for the induction of innate immunity, activation of both MyD88 and TRIF has been demonstrated following virus infection [75] and often induces an opposite effect on inflammatory gene expression [76]. Growing reports describe the effect of TLR adaptors namely MyD88 and MyD88-adaptor-like (MAL)/TIRAP are involved in the negative regulation of alternative TLRs [73, 74, 77, 78]. It has been reported that although MyD88 activates all TLRs except TLR3, MyD88 also functions as a negative regulator of TLR3. In vitro studies suggest that in addition to TLR3, both RIG-I and MDA-5 also detect RNA viruses or analogues (e.g., poly I: C) and activate IRF-3 and IRF-7 which results in the induction of the antiviral IFN-β [79]. Therefore, not only TLRs but also RLRs may work together or independently through the recruitment of adaptor proteins MyD88 or TRIF in perpetuation of downstream signaling cascades for mounting a productive immune response [79]. However, during many viral infections, for example, Coxsackievirus B3, VEEV, or Marburg virus infection which also utilizes RLR signaling pathways, a significant upregulation MyD88 has been observed [80–82]. This increase in MyD88 upregulation and interaction of MyD88 with IRF3/IRF7 in sequestering away IRF3/IRF7 has been proposed to exert an inhibitory effect on TLR3- or TRIF-mediated downstream signaling pathway of type I IFN response. Thus, MyD88 interaction with IRF3/IRF7 is linked to weak immune signaling and curtail IFN-β induction which is critical at the early stage in clearing the infection [73, 74, 83]. These data are consistent that IFN-β gene induction in MyD88- and Mal/TIRAP-deficient cells that were significantly enhanced following poly I:C (dsRNA) stimulation, or upon treatment of wild-type cells with Mal/TIRAP-inhibitory peptide [73, 74]. Largely, these reports suggest that significant upregulation of TIR domain–containing adaptors such as MyD88 and Mal/TIRAP also negatively regulate the IFN-β induction which is essential for antiviral effect [73, 74]. In support of the data, MyD88^−/−^ mice were shown to have a significant higher survival rate (86%) in contrast to MyD88^+/+^mice (35%) after CVB3 infection or HSV-1 [71, 80]. Also, in MyD88^−/−^ DCs compared to the responses in MyD88^+/+^ DCs following exposure to EBOV virus-like particles (eVLPs), a significant increase in IFN α/β, IRF1, and IRF7 along with increased expression of interferon-stimulated genes (ISG) observed [84]. Basically, these reports suggest that MyD88 upregulation impairs the type I IFN response during many virus infections. Studies from our laboratory have reported that limiting sequestration of IRF3/IRF7 in the presence of a small molecule inhibitor of MyD88 resulted an increase in type I IFN response, suppression of viral replication, and improved survival, weight change, and clinical disease scores in mouse model of viral diseases [83]. MyD88 inhibition concurrent with an increase in phosphorylation IRF-3 was consistent with an increase in the type I IFN response which is indicative of alternate TRIF-IRF3-axis–mediated IFN-β induction.

### 2.1 MyD88 as a potential therapeutic target for tangling severe inflammation and augmenting antiviral type I IFN response with SARS-CoV-2 (COVID-19) infection: a proposition for post-exposure therapy against COVID-19.

**Fig. 3:**
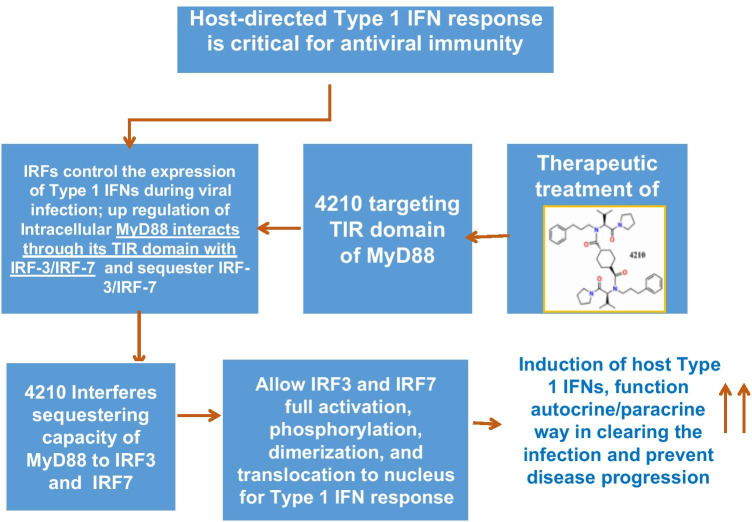
Plausible mechanism of antiviral type I response by MyD88-targeted therapy

Since the discovery of MyD88, a considerable progress has been made on the understanding of MyD88-linked antiviral type I IFN response and other pro-inflammatory cytokine responses with many viral and bacterial infections including its spatiotemporal regulation and function. The consequence of early strong and effective innate immune responses largely leads to overall host immunity including activation of various immune cells. The balance between host immune control and viral immune evasion is pivotal to viral pathogenesis. An unbalanced immune response, characterized by a weak production of type I IFNs and an intensified release of unbalanced pro-inflammatory cytokines, contributes to the severe forms of the many viral diseases including the current COVID-19 pandemic with the severe acute respiratory syndrome coronavirus-2 (SARS-CoV-2) infection [85, 86]. The modest IFN response could explain why viremia peaks at early stages of the disease, at the time of symptoms appearance, and not around 7 to 10 days following symptoms, like during SARS-CoV and MERS-CoV infections. Also markedly elevated plasma levels of pro-inflammatory cytokines including IL-6 and chemokines have been detected in patients with COVID-19 [87, 88], associated with severe pathology and impaired lung functions. While it is known that dysregulation/over-activation of MyD88 contributes to exacerbated inflammatory response, the so-called cytokine storm, and in patients with SARS-CoV-2 infections, provides evidence that SARS-CoV-2 pathogenesis is at least partially controlled by innate immune signaling, although it is yet undetermined whether upregulation of MyD88 is linked with impaired type IFN response with SARS-CoV-2 infection. Recent research highlights that key components of innate immune signaling pathways including host factor such as MyD88 regulate exacerbated inflammatory response [83]. It has also been reported that with many virus infections, upregulation of MyD88 is associated with the impairment type IFN response, and MyD88 inhibition improved type IFN response and suppressed virus replication and improved survival in a mouse model of multiple viral diseases [80, 83, 89–91]. Generally, the balance between the host type I IFN induction and the ability of virus to spread is determined in the first few hours of virus replication. While viral proteins are known to exert an effect on the interference of type I IFN induction and evade host antiviral innate immune effector mechanism, further detailed mechanistic studies are required to determine whether the IFN antagonists are identified in SARS-CoV-2 [85]. Also, earlier studies highlight the importance of TLR adaptor signaling in generating a balanced protective immune response to highly pathogenic SARS coronavirus infections and in particular TRIF-mediated immune signaling contributes to a protective innate immune response [86]. Therefore, it is yet unknown whether upregulation of intracellular host cell factor(s) included MyD88 which is the convergence point of pro-inflammatory signaling pathways, responsible for severe pro-inflammatory cytokines while dampening the antiviral type I IFN response through sequestration of IRFs from TRIF pathways. A strong type I IFN induction at the early stage of infection is indispensable for vertebrates to control viral infections because modulation of innate immune responses in a balanced manner promotes antigen presentation and natural killer cell functions [92]. Impaired T cell response leads to lymphopenia, and functional exhaustion of CD4^+^ and CD8^+^ T cells is associated with COVID-19 [93] which can result from IFN production deficiency. IFNs are also known to be important regulators of the development of regulatory T cells (T_reg_), and T_reg_ cell counts in patients with COVID-19 have been shown to inversely correlate with disease severity [87, 94]. While dysregulated MyD88-mediated pro-inflammatory signaling leads to cytokine storm, the production of antiviral type I IFNs is reportedly blunted; thus, targeting MyD88 in limiting the inflammatory response and allowing more TRIF-mediated immune signaling for augmenting antiviral type I IFN response would be highly desirable. Although small molecule identified with type 1 IFN–inducing properties by high-throughput screening has been reported, however, the molecular target and the mechanism-associated IFN induction is yet unknown (Fig. 3) [95]. Our laboratory demonstrated that a lead inhibitor of MyD88, compound 4210 functions as an immune modulating molecule through deactivation of MyD88, adjusts biological pathways and accelerates type I and potentially type III IFN signaling centered on the activation of IRFs for strong host antiviral IFN response [83]. Similar to compound 4210, other small-molecule inhibitors of MyD88 (blocker of TIR-domain-homodimerization of MyD88) such as T6167923 and S5 [96, 97] have also shown type I–inducing properties (unpublished data). Since imbalanced host response to SARS-CoV-2 drives the development of severity of COVID-19 disease [88], it is anticipated that pharmacologic inhibition of MyD88 would likely induce an antiviral type I IFN as well as providing TIR blockade in limiting pro-inflammatory cytokines. Thus, MyD88-targeted disease-modifying therapy tangling these two-pronged antiviral mechanisms may be a potential post-exposure therapy in tackling COVID-19 disease.

### Conclusions and future directions

The proof-of-concept on MyD88-targeted therapeutic approach in controlling inflammatory diseases has been validated, and even first phase clinical trial against COPD was proved to be successful [51]; reviewed by Padova et al.]. Also, recent research revealed underlying mechanisms of MyD88 in the impairment of antiviral type IFN signaling through MyD88-IRF interaction which influences MyD88-independent alternate pathways of immune signaling with many virus infections. Recent report on therapeutic inhibition of MyD88 in restoring host antiviral type IFN response in MyD88-inpedendent pathways has been described in vitro with several viral infections and also in mice models of viral diseases. These encouraging data suggest that host-directed therapy in enhancing the host immune response is feasible by stimulating mechanisms that are involved in host defense, particularly target pathways that are perturbed as a consequence of pathogen exposure.

In addition, conventional antimicrobials that directly target pathogens must continue, but additional alternate approaches are also required particularly in the absence of broad-spectrum therapeutics or vaccination strategies effective for bacterial and viral infections. Development of novel host-targeted therapeutic treatment models, a complementing strategy with small molecules targeting host factors, offers a benefit for broad-spectrum host-directed therapeutic approach. Over the years, the development of such therapeutic agents targeting the host factor(s) that are critically involved in regulating the function of the immune system and/or other cellular processes are very limited. The critical barrier toward the development of such host-directed therapeutic is the identification and/or validation of appropriate therapeutic target(s) that play a critical role during a broad range of emerging/re-emerging as well as opportunistic viral/bacterial infections. In the case of targeting hyper-inflammation and imbalanced immune responses, treatment should be focused on symptomatic rather than causal, which likely reduces exacerbated tissue damage in infectious diseases. Therefore, targeting host factor critical regulating host overall immunity would likely enhance the immune response by stimulating mechanisms that are involved in host defense against the pathogen, particularly target pathways that are perturbed as a consequence of pathogen exposure and contribute to hyper-inflammation that lead to dysbalanced responses at the site of pathology [98]. In addition, anti-infectives that directly target the pathogen, adjunct therapy targeting host factors such as MyD88, a central component in immunological pathways, in the induction or adjusting host immunity will add an extra advantage. A short-term treatment with MyD88 inhibitors combined with canonical anti-infectives will provide untapped opportunities for restoring overall host immunity.

Attenuation of the MyD88 signaling pathways as an anti-inflammatory strategy is clearly beneficial. In this review, our discussion focused mostly on MyD88, and its role in the regulation of host-immune signaling pathways in the context of reducing exacerbated inflammation to enhance immunomodulation and/or balance host reactions at the site of pathology likely holds promise for the selective and symptomatic treatment of infectious diseases. Based on the available published evidence, the role of immune response to SARS-CoV-2 infection indicates that the host immune response plays an important role in controlling SARS-CoV-2 infection and the immune dysregulation can significantly modify the clinical outcomes of affected patients [99]. Recently, Hadadji et al. reported that impaired type I IFN activity (characterized by no IFN-β and low IFN-α production) and exacerbated inflammatory responses in severe COVID-19 patients [100] provide insights into the treatment of severe COVID-19 through induction/adjusting host type I IFN response. The concept of targeting a major host factor such as MyD88 affecting dual pathway directly such as exacerbated inflammation-causing lung pathology and respiratory distress and indirectly restricting antiviral type I IFN response is certainly acceptable. Modulation of host immunity to clinical outcome particularly those with pandemic potential may have a great therapeutic value in particular to COVID-19. Recent reports showed that patients treated with IFN-alpha -2b did not show evidence of a cytokine storm, one of the dangerous responses observed in some COVID patients [101]. In experimental animal model, treatment with MyD88 inhibitor increased IFN-β and reduced inflammatory cytokine response which showed promising results likely achieving these dual goals. Targeting the host factors such as MyD88 and pathways in innate immune modulation such type I IFN and also type III IFN to restrict productive replication virus and spread offers the opportunity for broad-spectrum antiviral drugs. But more in-depth academic and pharmaceutical research is required for the successful developments of inhibitors of MyD88 dimerization.

Taken together, the data presented in this review suggest that the pharmacological blockade with a short-term treatment with MyD88 inhibitors showed type I IFN induction while reducing acute exacerbation of various inflammatory cytokines. Given that overall imbalance in host immune system with inflammatory diseases including toxic shock, COPD as well as COVID-19 which is more to do with controlling inflammation, MyD88-targeted therapy would likely provide an advantage in treating severe inflammatory responses. At present, available data suggest the benefit of MyD88-targeted therapy with broad-spectrum antiviral type I IFN inducing properties; future in-depth efforts should be focused for the suitability of this approach in deploying to complex diseases like COPD and COVID-19 in limiting inflammation-associated syndrome to infection.

## Declarations

### Disclaimer

*Army*: Views expressed in this paper are those of the author and do not purport to reflect official policy of the US Government or USAMRIID/ARL administrators.

Opinions, interpretations, conclusions, and recommendations are those of the author and are not necessarily endorsed by the US Army.
